# Sarcome myéloïde orbitaire bilatérale: circonstance de découverte d´une leucémie aiguë myéloïde (à propos d´un cas)

**DOI:** 10.11604/pamj.2021.39.145.25242

**Published:** 2021-06-23

**Authors:** Rado Rambeloson, Njara Francia Ranoasy, Ony Randrianjafisamindrakotroka, Andoniaina Orlando Andriamiadanalisoa, Léa Raobela

**Affiliations:** 1 Centre Hospitalier Universitaire Ravoahangy, Antananarivo, Madagascar

**Keywords:** Sarcome myéloide, exophtalmie, Madagascar, à propos d’un cas, Myeloid sarcoma, exophthalmos, Madagascar, case report

## Abstract

Le sarcome myéloïde est une forme rare parmi les manifestations oculaires au cours de la leucémie aiguë myéloïde. Le caractère bilatéral et précédant tout signe clinique de la leucémie est d´autant plus rare. Nous rapportons le cas d´un patient de 16 mois consultant pour une exophtalmie aigue bilatérale d´allure inflammatoire, rapidement progressive, avec une kératite d´exposition. La tomodensitométrie oculo-cérébrale montrait une infiltration d´allure tissulaire bilatérale occupant la cavité orbitaire. Le bilan sanguin montrait un syndrome inflammatoire biologique, une bicytopénie et une blastose circulante à 83%. La présence de blastes circulant a motivé la réalisation d´un myélogramme qui a permis d´établir le diagnostic de leucémie aiguë myéloïde 4. Devant une exophtalmie, même bilatérale, il faut penser au sarcome myéloïde et étudier minutieusement les résultats de l´hémogramme.

## Abstract (en)

Myeloid sarcoma is a rare ocular manifestation of acute myeloid leukemia. Bilateral manifestation preceding any clinical signs of leukemia is even rarer. We here report the case of a 16-month-old patient with rapidly progressive, inflammatory, acute bilateral exophthalmos associated with exposure keratitis. Computed tomography (CT) scan of the orbit and of the brain showed bilateral tissue-like infiltration into the orbital cavity. Blood tests showed biologic inflammatory syndrome, bicytopenia and circulating blasts 83%. Myelogram was performed based on the presence of circulating blasts; it showed acute myeloid leukemia (grade 4). Clinicians should suspect myeloid sarcoma in patients with bilateral exophthalmos and conduct a careful interpretation of blood test results.

## Introduction

Le sarcome myéloïde est une tumeur extramédullaire composée de cellules immatures. Elle est aussi appelée sarcome granulocytique ou encore chlorome à cause de sa coloration verdâtre due à l´exposition de l´enzyme myélopéroxydase aux ultraviolets. Toutefois cet aspect n´est pas obligatoire, en effet, 30% des sarcomes myéloïdes n´ont pas de coloration verte. Le sarcome myéloïde a été inclus dans la classification de l´Organisation Mondiale de la Santé comme un des sous-groupes majeurs des cancers myéloïdes et leucémies [1]. Il peut apparaitre au cours d´une myélodysplasie, l´acutisation d´une leucémie myéloïdes chronique mais surtout au cours d´une leucémie myéloïde aigue. Sa survenue peut être inaugurale, synchrone ou révélatrice d´une rechute [2]. Le sarcome myéloïde est une forme rare parmi les manifestations oculaires au cours de la leucémie aiguë myéloïde. L´aspect est celui d´une exophtalmie uni ou bilatérale rapidement progressive avec des signes inflammatoires évoquant souvent une cause inflammatoire ou infectieuse. Ainsi, le diagnostic peut s´avérer difficile en absence de signes systémiques évidents de leucémie aiguë myéloïde.

## Patient et observation

**Informations sur le patient**: nous rapportons le cas d´un patient de 16 mois emmené par ses parents pour une exophtalmie bilatérale rapidement progressive datant de 2 semaines avant la consultation. Le patient n´avait pas d´antécédents particuliers, les parents avaient affirmé l´absence de traumatisme. L´anamnèse avait permis de retrouver un chémosis d´emblée bilatéral ayant évolué brutalement vers une exophtalmie inflammatoire avec baisse de la vision, cela précède d´un syndrome grippal.

**Résultats cliniques**: l´examen avait retrouvé une acuité visuelle réduite à une perception lumineuse mal orientée. Une exophtalmie bilatérale non axile, douloureuse, non réductible et non vasculaire a été objectivée. A cela était associé un œdème palpébral bilatéral, un chémosis annulaire, une kératite d´exposition et une ophtalmoplégie, l´examen du fond des yeux était impossible à cause de l´état des cornées.

**Évaluation diagnostique**: la tomodensitométrie oculo-cérébrale montrait une infiltration d´allure tissulaire bilatérale occupant la cavité orbitaire. Le bilan sanguin montrait un syndrome inflammatoire biologique, une bicytopénie et une blastose circulante à 83% (Tableau 1). La présence de blastes circulant a motivé la réalisation d´un myélogramme qui a permis d´établir le diagnostic de LAM4 variante éosinophile selon la classification FAB (Tableau 2). Le prélèvement in situ de l´infiltrat tissulaire n´a pas été fait étant donné la thrombopénie. Ainsi, le diagnostic de sarcome myéloïde a été retenu sur les données cliniques ophtalmologiques, l´hémogramme et le résultat du myélogramme.

**Tableau 1:** résultat de l´hémogramme

Variables	Résultats
VSH	120
Globules rouges	7,8 g/dL
globules blancs	189 G/L
PNN	10
PNO	00
PNB	00
Lymphocytes	06
Monocytes	01
Autres cellules	83% blastes
Plaquettes	73 G/L

**Tableau 2:** résultat du myélogramme

Lignée granuleuse	19%
Myélocytes neutrophiles	04%
Polynucléaires neutrophiles	06%
Polynucléaires éosinophiles	09%
**Lignée érythroblastique**	**02%**
Proérythroblastes	01%
Erythroblastes basophiles	01%
**Lymphocytes**	**01%**
**Blastes**	78% Blastes de tailles moyenne à grandes. Noyaux à chromatine fine, nucléolés. Cytoplasme basophile, très granuleux RNC élevé. Myéloblastes 56% Monoblastes 22%
**Interprétation**	Aspect cytologique en faveur d´une leucémie aiguë myélo-monocytaire avec des éosinophiles anormaux, LAM4 variante éosinophie selon la classification FAB

**Intervention thérapeutique**: le patient a été perdu de vue après l´annonce du diagnostic.

## Discussion

La leucémie aiguë myéloïde est une maladie relativement rare mais représente une grande proportion parmi les décès par cancers. C´est la plus fréquente des leucémies chez l´adulte. Elle est plus rare chez l´enfant. La leucémie représente environ 30% des cancers de l´enfant. La leucémie aiguë myéloïde est plus rare que la leucémie aiguë lymphoïde, elles représentent respectivement 18 et 82% des leucémies aiguës chez l´enfant [3]. La leucémie aiguë myéloïde présente 2 pics d´incidence : chez l´enfant et chez les personnes âgées. Selon Howlader, l´incidence chez l´enfant de 0 à 14 ans était de 7,7 nouveau cas par million d´enfant entre 2005 et 2009. Chez les enfants de moins de 1 an, elle est de 18 nouveaux cas par million d´enfants [4]. Au moment du diagnostic, un enfant sur deux présente une atteinte oculaire. Le patient peut être asymptomatique ou se plaindre d´une baisse de vision. Il s´agit surtout de complications liées à l´insuffisance médullaire et à l´hyperviscosité du sang. L´infiltration oculo-orbitaire de cellules blastiques est beaucoup moins fréquente [5]. Sachat, sur 120 patients constate 42% de manifestations oculaires et parmi ces 42% seulement 3% ont une infiltration par des cellules blastiques, 39% ont une atteinte secondaire aux complications de la maladie [6].

L´expression clinique d´un sarcome granulocytique orbitaire est une exophtalmie d´allure inflammatoire uni ou bilatérale avec un chémosis annulaire d´installation rapidement progressive. Une exophtalmie bilatérale isolée révélant une leucémie aiguë est peu commune. La plus large série étudiée concernant des enfants était composée de 33 cas avec seulement 4 exophtalmies bilatérales initiales avec une prédominance masculine [7]. Le plus jeune cas récemment rapporté avec une exophtalmie bilatérale en tant que manifestation initiale d´une leucémie aiguë myéloïde est un enfant de sexe masculin âgé de 19 mois [8]. Ce qui fait de notre patient le plus jeune cas rapporté à notre connaissance. Le cas de notre patient rappelle la difficulté étiologique devant une exophtalmie aiguë, d´autant plus qu´elle est bilatérale. Devant un tel tableau, le diagnostic différentiel est vaste. Parmi cela, on peut citer une ophtalmopathie disthyroidienne, une cellulite orbitaire bilatérale, une thrombose du sinus caverneux, une extension orbitaire d´un rétinoblastome bilatérale, un rhabdomyosarcome, une pseudotumeur orbitaire, un gliome du nerf optique.

Devant toutes ces possibilités, les examens morphologiques sont nécessaires pour différencier les lésions pouvant avoir un même aspect clinique. Pour notre patient, l´imagerie par résonnance magnétique n´étant pas accessible, la tomodensitométrie reste un examen qui garde sa place bien que relativement peu spécifique. L´aspect tomodensitométrique d´un sarcome granulocytique est une masse hétérogène, dense, extensive, envahissant les structures avoisinantes. Chez notre patient, la tomodensitométrie associée au bilan clinique stérile mise à part l´exophtalmie n´était pas suffisants pour orienter le diagnostic. Ils avaient plutôt orienté vers une cellulite orbitaire bilatérale. Ce sont les données de l´hémogramme qui ont permis d´orienter le diagnostic en retrouvant une anémie à 7,8 g/dL, une thrombopénie à 73 G/L et une hyperleucocytose avec 83% de blastes circulants. En effet une blastose circulante supérieure à 5% et un médulogramme qui retrouve une moelle riche avec une blastose supérieure à 30% confirme l´origine hématologique du syndrome tumoral. L´étude cytologique et cytochimique des blastes détermine le sous type de leucémie aiguë qui était une LAM4 variante éosinophile selon la classification FAB (French–American–British classification) chez notre patient. Cette variante figure parmi les sous types pourvoyeur de sarcome granulocytique [9].

Le diagnostic de certitude peut être obtenu par la biopsie de la masse tumorale. La coloration à l´hématoxyline et à l´éosine met en évidence le plus souvent une infiltration diffuse de cellules granuleuses et de myéloblastes. Les cellules tumorales ont un cytoplasme abondant avec un noyau large. En immunohistochimie, le chlorome est identifié par son immunoréactivité pour CD33, CD34, CD43, CD117, CD68, et la myéloperoxydase. Cependant, la plupart des auteurs soulignent la difficulté du diagnostic histologique, le diagnostic peut ne pas être retrouvé dans 50% des cas. D´où l´intérêt de l´hémogramme et du myélogramme qui permettent d´entreprendre rapidement le traitement. Effectivement Murthy avait étudié une série de 12 patients présentant une exophtalmie, par la suite, chez 8 d´entre eux il avait diagnostiqué une leucémie aiguë myéloïde et le diagnostic a été retenu après uniquement des examens sanguins [10]. Toutefois, dans certains cas l´examen hématologique et le myélogramme peuvent ne pas suffire car la manifestation oculo-orbitaire peux précéder la maladie de plusieurs mois. Dans ces cas-là l´examen histologique est indispensable.

**Point de vue du patient**: le patient a été perdu de vue peu après l´annonce du diagnostic.

**Consentement éclairé**: les parents du patient a été informé sur la pertinence du dossier et sur l´utilisation ultérieure du dossier.

## Conclusion

Devant une exophtalmie aiguë bilatérale chez le nourrisson, même en absence de manifestations cliniques de leucémie aiguë, il faut inclure le sarcome myéloïde dans les diagnostics à évoquer et ainsi orienter les examens paracliniques, voire pratiquer un myélogramme en cas de doute.
